# Biological Characterization and Essential Oil Profiles of Eastern European Cultivars of *Thymus*, *Satureja*, and *Monarda*

**DOI:** 10.3390/molecules31020338

**Published:** 2026-01-19

**Authors:** Katarzyna Pokajewicz, Monika Chodura, Hayet Ahlem Lezrag, Liudmyla Svydenko, Małgorzata Nabrdalik, Ewa Moliszewska, Sofiane Fatmi, Nataliia Hudz, Piotr P. Wieczorek

**Affiliations:** 1 Institute of Chemistry, University of Opole, 45-052 Opole, Polandnataliia.hudz@uni.opole.pl (N.H.); pwiecz@uni.opole.pl (P.P.W.); 2 Associated Laboratory in Marine Ecosystems and Aquaculture, Faculty of Nature and Life Sciences, Université de Bejaia, Bejaia 06000, Algeria; hayetahlem.lezrag@univ-bejaia.dz (H.A.L.); sofiane.fatmi@univ-bejaia.dz (S.F.); 3 Institute of Climate-Smart Agriculture of the NAAS of Ukraine, Khlibodarske, Odesa District, 67667 Odesa, Ukraine; svid65@ukr.net; 4 Institute of Environmental Engineering and Biotechnology, University of Opole, 45-040 Opole, Poland; mnabrdalik@uni.opole.pl (M.N.); ewamoli@uni.opole.pl (E.M.); 5 Department of Drug Technology and Biopharmacy, Danylo Halytsky Lviv National Medical University, 79010 Lviv, Ukraine

**Keywords:** *Thymus*, *Satureja*, *Monarda*, essential oil, antimicrobial activity, toxicity, spermatozoa, GC, chemotype, phenolic monoterpenes

## Abstract

*Thymus*, *Satureja*, and *Monarda* are three plant genera, belonging to the Lamiaceae family, that are particularly valued for their essential oils (EOs) abundant in phenolic terpenoids. In this study, a botanical characterization of the following eight Eastern European cultivars (some of them newly bred) grown in Ukraine is distinguished: *Thymus vulgaris* (‘Yalos’, ‘101’), *Thymus richardii* (‘Fantasia’), *Satureja montana* (‘Krymsky smaragd’, ‘Lunata’, ‘4-18’), *Monarda fistulosa* (‘Premiera’), and *Monarda* × *hybrida hort.*, which is a *Monarda didyma* × *Monarda fistulosa* hybrid (‘Tonya’). The EO of those cultivars was obtained and characterized in detail using GC-MS and GC-FID. Additionally, some biological activities of these oils were tested. Antimicrobial activity was verified against *Escherichia coli*, *Staphylococcus aureus*, and *Candida albicans* using disk diffusion and microdilution methods. Furthermore, some preliminary tests were performed on the motility of bull sperm using the CASA system. All the *Thymus* cultivars were very rich in thymol (57.99–67.62%), and all the *S. montana* cultivars were very abundant in carvacrol (62.22–75.53%). *M. fistulosa* cv. ‘Premiera’ contained mainly thymol (49.87%), and *M.* × *hybrida* cv. ‘Tonya’ contained both thymol (46.70%) and carvacrol (10.37%). All the tested EOs, as well as thymol and carvacrol, exhibited strong antibacterial and antifungal action with minimal inhibitory concentrations ranging from <0.25–0.5 mg/mL for *Satureja*, through <0.25–0.5 mg/mL for *Monarda*, to 0.5–4 mg/mL for *Thymus*. The EOs, at a concentration of 0.4 µL/mL, exhibited cytotoxicity towards bull spermatozoa when compared to the control sample.

## 1. Introduction

*Thymus*, *Satureja*, and *Monarda* are three popular plant genera belonging to the Lamiaceae (mint) family. They belong to the same tribe—*Mentheae*—but phylogenetically their lines of evolution were split in the past [1]. *Thymus* and *Satureja* are more closely related and are native to the Mediterranean area, while *Monarda* is endemic to North America [1,2,3]. These plants were used in traditional medicine across various cultures for treating a wide range of conditions [2,3,4,5]. Nowadays, the plants are used in many applications in everyday life and different industries, with the main prominent use in culinary (for *Thymus* and *Satureja*), whereas *Monarda* seems to be more commonly used as an ornamental plant in gardens.

The crucial raw material obtained from the above-discussed aromatic plants is their essential oil (EO). These EOs are rich in monoterpenes, including phenolic compounds such as thymol and carvacrol. Their chemical profile varies and depends on the actual species and chemotype but often contains a large share of thymol and carvacrol, which exhibit potent antimicrobial and antioxidant properties, thus making them valuable for use in food preservation, cosmetics (especially in oral care), herbal medicines, and biodegradable and natural-based pesticides [2,3,6].

In the past, in Ukraine, the aromatic plant plantations were located mostly on the Crimean Peninsula. Due to the need to relocate this cultivation from annexed Crimea to other regions of Ukraine, the Ukrainian researchers from Institute of Rice of the Ukrainian National Academy of Agrarian Sciences (NAAS) worked on introducing of various cultivars of aromatic plants, as well as breeding new ones and their use that would be adapted to the harsher conditions of the Ukrainian steppe (periodic droughts and large daily temperature fluctuations—higher resistance to frost and fungal infections) and enhanced decorative features (longer flowering period, more abundant flowering, beautiful bush habit, etc.) [7]. This study presents the botanical description, chemical characteristics, and preliminary screening of biological activities of selected adapted and newly bred cultivars of the following species: *Thymus vulgaris*, *Thymus richardii*, *Satureja montana*, *Monarda fistulosa*, and *Monarda* × *hybrida hort.* (*Monarda didyma* L. × *Monarda fistulosa* L.), which were adapted and developed during these works.

Genus *Thymus* contains 272 accepted species [8] and is one of the largest genera of the Lamiaceae family [9]. *Thymus vulgaris* L., commonly known as thyme, is the most widely recognized, used, and studied species within the genus. It is an evergreen herb that is native to Spain, the Balearic Islands, France, and Italy [8,10]. The thyme’s EO contains terpenes and terpenoids such as thymol, carvacrol, linalool, geraniol, α-terpineol, 1,8-cineole, *p*-cymene, borneol, myrcene, γ-terpinene, 4-thujanol, caryophyllene, and caryophyllene oxide [3,10,11]. The exact chemical composition may vary even within the same species, despite having similar morphology [11]. The highest proportion of a given component defines its chemotype, and multiple different chemotypes of *T. vulgaris* are usually distinguished: thymol, carvacrol, linalool, geraniol, 1,8-cineole, α-terpineol, and sabinene hydrate (thujanol-4) [11]. The thymol chemotype is the most common. Thymol is responsible for the strong and pleasant aroma of the EO. Thymol and carvacrol are usually accompanied in EOs by other monoterpenes, most frequently γ-terpinene or *p*-cymene, which represent, respectively, the precursor and the alternative product formed in the biosynthetic pathway leading to carvacrol and thymol synthesis [11,12].

*Thymus richardii* Pers., the second species of *Thymus* studied, is much rarer than *T. vulgaris*. It is native to Sicily, Spain, and the Balkans [8,9,13,14]. It contains five subspecies, and *T. richardii* subsp. *nitidus* (Guss.) ‘Yalos’ is endemic to the island of Sicily [8,9]. The EO of *T. richardii* is known to contain mostly *p*-cymene, γ-terpinene, thymol, carvacrol, methyl thymol ether, β-bisabolene, caryophyllene, caryophyllene oxide, germacrene-D, 1-terpinen-4-ol, and camphene [13,14].

The *Satureja* genus includes 45 accepted species and is native to southern and southeastern Europe, northern Africa, the Middle East, and Central Asia [8]. *Satureja montana* L. is one of the most popular species. It is a perennial shrub commonly known as winter savory. Its EO is abundant in monoterpenes, including carvacrol, thymol, *p*-cymene, γ-terpinene, linalool, and terpinen-4-ol. The largest quantitative share is usually constituted by carvacrol, thymol, their methyl ethers, and *p*-cymene, followed by sesquiterpenes [15,16,17]. Similarly to thyme, different chemotypes have been identified, including phenolic chemotypes with predominant carvacrol or thymol, as well as *p*-cymene, linalool, geraniol, and *cis*-sabinene hydrate. Apart from the genetic factors, the content of individual compounds varies depending on the region and climatic conditions [18].

Genus *Monarda*, as mentioned above, represents the North American branch of the mint family plants. According to Plants of the World Online, which is a botanical and taxonomic database maintained by the Royal Botanic Gardens in Kew (London), it includes 25 accepted species [8]. *Monarda fistulosa* (wild bergamot) and *Monarda didyma* L. (Oswego tea) are some of the most popular species of this genus. *Monarda* × *hybrida hort*. (*Monarda didyma* L. × *Monarda fistulosa* L.) is a hybrid of *Monarda fistulosa* and *Monarda didyma* that is grown in gardens but still lacks officially registered taxonomic status [8]. The EOs of *Monarda* species are rich in oxygenated monoterpenes, especially thymol and carvacrol, but also include chemotypes with domination of linalool and geraniol [19,20,21].

The biological activity of the plant species described above is mostly linked to the phenolic monoterpenes such as thymol and carvacrol, which have a vast spectrum of biological activities, confirmed in multiple in vitro and in vivo studies [22,23,24,25,26,27]. The EOs, which are rich in those components, such as those from *Thymus*, *Satureja*, and *Monarda*, possess interesting properties that can be utilized in numerous applications across various pharmaceutical, food, cosmetic, and agricultural industries. Due to their combination of both antimicrobial and antioxidant properties, thymol- and carvacrol-rich EOs are promising natural bioactive preserving agents for food as well as biological specimens, such as semen used in animal reproduction. Therefore, the goal of this study was to provide botanical characterization of adapted and newly bred Eastern European cultivars grown in the Kherson region, along with a comprehensive chemical characterization of their EOs, and to preliminarily evaluate their antimicrobial and cytotoxic activities.

## 2. Results and Discussion

### 2.1. Botanical Characterization

#### 2.1.1. *Thymus*

**Figure 1:**
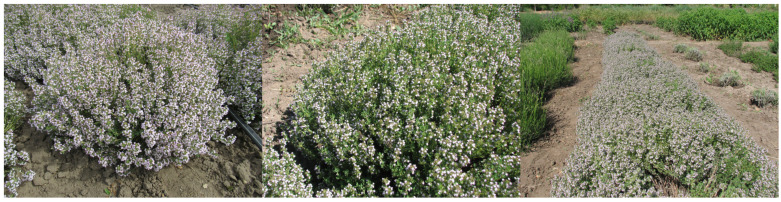
Photographs showing (from left) studied plants, respectively: *Thymus vulgaris* L. cultivar ‘Yalos’; *Thymus vulgaris* L. cultivar ‘101’; *Thymus richardii* cultivar ‘Fantasia’; photo by Liudmyla Svydenko.

*Thymus vulgaris* L. cultivar ‘Yalos’ (Figure 1) is an evergreen compact shrub that is 25–30 cm in height and 70–80 cm in diameter. The flower-bearing stems are lignified in the lower part and branched. The leaves are small, densely pubescent on both sides, short-petiolate, oblong-lanceolate, grayish green, 7–8 mm long and 2–2.5 mm wide. The leaf margins are bent inwards. The flowers are small, purple-pink, and arranged in an elongated, intermittent inflorescence consisting of 5–6 rings. There are 16–20 flowers in a ring. Seeds are small, round, and dark brown. Frost resistance is 7 points. In the Kherson region, the usual mass fraction of EO is 0.4–0.45% of the crude weight. Sample 500 was harvested at the beginning of full flowering with a 0.3% yield of EO.

*Thymus vulgaris* L. cultivar ‘101’ (Figure 1) is an evergreen shrub, 30 cm tall and 80 cm in diameter. Flowering stems are lignified in the lower part and branched. The leaves are small, 7–8 mm long, 2.0–2.5 mm wide, densely pubescent on both sides, short-petiolate, oblong-lanceolate, and gray-green. The leaf margins are turned inwards. The flowers are small, purple-pink, and collected in an elongated, intermittent inflorescence consisting of 5–6 rings. There are 16–20 flowers in a ring. Flowering is more abundant than that of ‘Yalos’. Resistance to frost is also higher than that of ‘Yalos’ (9 vs. 7 on the bonitation scale). In the Kherson region, the usual mass fraction of EO is 0.35–0.4% of the crude weight. Sample 503 was harvested during full flowering and produced a 0.6% yield.

*Thymus richardii* subsp. *nitidus* cultivar ‘Fantasia’ (Figure 1) is an evergreen shrub that is 40–45 cm tall and 65–70 cm in diameter. The flower-bearing stems are lignified in the lower part and branched. Leaves are small, oblong-elliptic, densely pubescent on both sides, short-petiolate, 6 mm long, 4 mm wide, and grayish green. The leaf margins are strongly inwardly curled. The flowers are small, purple-pink, collected in an elongated spike-shaped intermittent inflorescence 4–8 cm long, consisting of 4–6 rings. There are 14–18 flowers in a ring. Corolla is pale purple in color. The seeds are small, rounded, and dark brown. In the Kherson region, the mass fraction of essential oil in the phase of mass flowering is usually 0.45–0.5% of the crude weight. Sample 502 was harvested during full flowering with a 0.7% yield.

#### 2.1.2. *Satureja*

**Figure 2:**
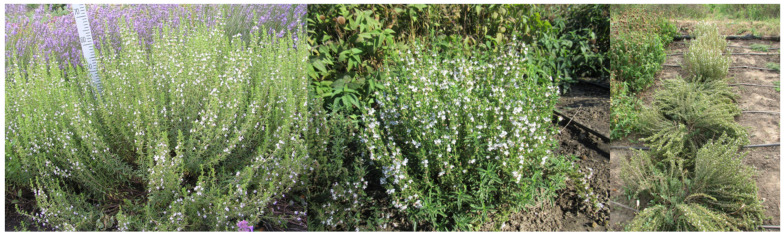
Photographs showing (from left) studied plants, respectively: *Satureja montana* L. cultivar ‘Krymsky smaragd’; *Satureja montana* L. cultivar ‘Lunata’; *Satureja montana* L. cultivar ‘4-18’; photos by Liudmyla Svydenko.

*Satureja montana* L. cultivar ‘Krymsky smaragd’ (Figure 2) is a perennial semi-shrub from the Lamiaceae family. Under the conditions of the steppe zone of southern Ukraine, the plants display a compact habit, growing to 50–55 cm in height with a spread of 80 cm. Each bush contains approximately 100 shoots. The stem is erect, almost round, densely leafy, with a branched upper part, and brown. The leaves are pointed, leathery, linear-lanceolate, 2.0–2.5 cm long and 0.5 cm wide, with punctate glands and dark green. The flowers are 1.0–1.3 cm in diameter, white with lilac dots on the petals of the lower lip and a lilac tinge on the edge of the upper lip. They are gathered in panicle-like inflorescences 22 cm long. The calyx is short, tubular, and the teeth are lanceolate and linear, almost twice as long as the tube. Plants begin mass flowering in the second decade of July. Sample 516 was harvested at full flowering, and the EO yield was 0.34%.

*Satureja montana* L. cultivar ‘Lunata’ (Figure 2) is a shrub reaching 40–42 cm in height and 60–70 cm in diameter, almost round. The stem is erect, densely leafy and branched at the top and light brown. The leaves are pointed, leathery, linear-lanceolate, 2.50–2.70 cm long and 0.40–0.50 cm wide and dark green. The flowers are large, with broad petals 1.3–1.5 cm in diameter, the flowers gathered in half-rings of 6–8 flowers. On the upper part of the stem, they form tassel-like inflorescences 17.8 cm long and 3.1 cm in diameter. The flower corolla is white. The calyx is short, tubular, and its teeth are stilted and linear, almost twice as long as the tubes. The onset of mass flowering is in the third decade of July, and the flowering time is 40–50 days. This variety is distinguished by its large white flower size, dense inflorescence, and late flowering period. It is drought- and winter-tolerant and has increased ornamental value due to its rounded bush shape and large flowers in the inflorescence. The mass fraction of EO in the phase of mass flowering is usually 0.45% by weight. Sample 517 was harvested at full flowering with a 0.36% EO yield.

*Satureja montana* L. cultivar ‘4-18’ (Figure 2) is a semi-shrub with a spreading habit, reaching a height of 45–50 cm and a diameter of 80–90 cm. The leaves are 2.0–2.1 cm long and 0.3–0.4 cm wide. The flowers are small, 0.9–1.1 cm in diameter, white with lilac dots on the petals of the lower lip, and gathered in a panicle-like inflorescence 27–29 cm long. The plant flowers early, with the onset of mass flowering observed in the first decade of July. The sample 518 was harvested at full flowering with an EO distillation yield of 0.22%.

#### 2.1.3. *Monarda*

**Figure 3:**
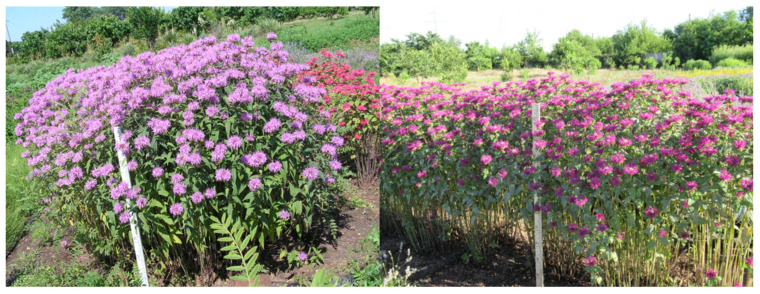
Photographs showing (from left) studied plants, respectively: *Monarda fistulosa* L. cultivar ‘Premiera’; *Monarda* × *hybrida hort*. cultivar ‘Tonya’; photos by Liudmyla Svydenko.

*Monarda fistulosa* L. cultivar ‘Premiera’ (Figure 3) is a perennial plant that in the steppe climate of Ukraine, reaches a height of 90–100 cm and a diameter of 55–60 cm. Young plants (2 or 3 years old) form 10 to 20 flowering stems. They have simple, serrated, dark green leaves (6.5–8.0 cm long and 3.0–3.8 cm wide). The flowers are small, clustered in black, spherical heads of pink color. Vegetation begins in the second or third decade of March, depending on the weather conditions of the year. Mass flowering occurs at the beginning of the third decade of June. Fruiting lasts from the third decade of July to the third decade of August. This cultivar is characterized by resistance to frost and pests. It grows well in sunny areas and in partial shade. It is decorative during flowering. It blooms for a long time and has a wonderful aroma. The EO content is on average 0.8–0.9% of the crude mass. Sample 512 was harvested at the end of flowering/beginning of fruiting and yielded 0.6% EO.

*Monarda* × *hybrida* cultivar ‘Tonya’ (Figure 3) is a first-generation hybrid that was created from seedlings obtained through free pollination between *Monarda fistulosa* and *Monarda didyma*. During the period of full flowering, the plants reach a height of 90 cm and a diameter of 60–70 cm, presenting a compact habit. They have simple, serrated, and deeply notched leaves of light green color with a dirty red shade, measuring 6.5 cm in length and 3.5 cm in width. The flowers are purple (beetroot color), and the diameter of the inflorescence is 6.0 cm. This variety is distinguished by decorative leaves, attractive flower color, and resistance to fungal diseases. The content of EO in this variety is usually around 0.45% of fresh plant mass. Sample 519 was harvested at the end of flowering/beginning of fruiting (yield 0.35%).

### 2.2. Chemical Profile of Essential Oil

#### 2.2.1. *Thymus*

In the studied thyme EOs, 60 different volatile components were identified and quantified (Table 1, Supplementary Material S1a). The main component for both *Thymus* species across all the studied cultivars was thymol at the level of 58% for cultivar ‘Yalos’ to 68% for cultivar ‘101’. Thus, the studied cultivars can be considered as a thymol chemotype. The second and third prevalent components were *p*-cymene and γ-terpinene, respectively. They, in turn, were the most abundant in the EO of cultivar ‘Yalos’, reaching 7% and 6%, respectively. These compounds were both considered as precursors in the synthesis of thymol [11,24,28]. The higher concentrations of *p*-cymene and γ-terpinene in this cultivar can be associated with its lower thymol content, suggesting a balance between substrate and product, which is a typical observation for this chemotype of thyme EO [29]. However, Krause et al. [12] presented the other mechanism of thymol/carvacrol biosynthesis, as formerly was assumed. They showed that γ-terpinene is oxidized by cytochrome P450 monooxygenases to produce unstable cyclohexadienol intermediates that were subsequently dehydrogenated by a short-chain dehydrogenase/reductase to form ketone intermediates. Subsequent keto–enol tautomerization leads to the formation of the aromatic monoterpenoids thymol and carvacrol. In this proposed mechanism, *p*-cymene is not the precursor of thymol/carvacrol but rather a by-product, resulting from spontaneous dehydratation of unstable dienol intermediates. The often observed occurring reverse correlation between *p*-cymene and thymol/carvacrol in EOs could be due to these competing metabolic pathways for unstable dienol intermediates.

The thymol content of 58–68% is quite high, representing the upper limit or even above the typical range of this compound in the EOs of the thymol chemotype. For example, Hudaib and coworkers have studied the EOs of *T. vulgaris* grown in Italy, collected and distilled in different periods of the plant’s vegetative cycle. The thymol content ranged from 19% to 54% [29]. In another article, Pluhár et al. described (after Kamondy) the observed variations in thymol content—from 14.0 to 60.27% in Hungarian EOs, depending on growing year and the time of harvest [30]. Satyal et al. [10] reported the thymol content of 47% in EO obtained from French-grown plants, while Galovičová et al. [31] found 48% in EO from cultivation in Slovakia, and Borugă et al. [32] reported the same value of 48% for Romanian thyme EO.

According to the European Pharmacopeia (Ph. Eur.), the allowed specification for thymol is between 37 and 55% [33]. The studied EO samples do not meet the limits, not only for thymol but also for other specified monoterpenoids, such as myrcene, α-terpinene, *p*-cymene, and partly γ-terpinene, which are, in turn, lower than the allowed limits. As mentioned above, γ-terpinene and *p*-cymene are related to thymol biosynthesis and often are negatively correlated. The dominant share of thymol in the EOs is due to the conversion of its precursor (γ-terpinene) to intermediate cyclohexadienol and the minimized competing pathway leading to *p*-cymene.

Similarly, according to ISO norm 19817 [34], which regulates the chromatographic profile of *T*. *vulgaris* and *T. zygis* EO (thymol type), the studied EOs contained too much thymol and too little *p*-cymene compared to the specified limits 

The high thymol content in the studied EOs most likely reflects a combination of cultivar features, growing conditions, and the plant’s phenological phase. However, as these factors were not examined within this study, the observed differences cannot be conclusively explained.

Another noteworthy component of the studied EOs is carvacrol (2.77–3.41%), with the highest concentration observed for cultivar ‘101’. The content of this compound is typical for thymol-type thyme EOs, and it is within the limits of Ph. Eur. Similarly, linalool, which was found to constitute 2.47–3.10% of the studied oils, also complies with Ph. Eur.

Compounds appearing in smaller amounts (in descending order) quantified in the studied EOs were *trans*-caryophyllene (1.13–2.91%), borneol (1.38–2.18%), caryophyllene oxide (1.04–1.47%), 1,8-cineole (1.04–1.49%), and *cis*-sabinene hydrate (0.44–1.89%). They are typical minor constituents of the thymol-type thyme EOs, and their presence is a natural result of the activity of secondary mono- and sesquiterpenes.

The EO of *Thymus richardii* is not as well characterized as those of other, more popular species such as *T. vulgaris* or *T. zygis*. In the literature, reports on its composition are rather limited, and none describe an EO containing as much as 63.69% thymol, as observed in our study.

Depending on the subspecies and the location of the plants, the EOs of *Thymus richardii* can be distinguished into different chemotypes: sesquiterpene (β-bisabolene) and monoterpene (also called phenolic, due to the presence of phenolic compounds and their related compounds). The first type is characteristic of plants growing in rocky, often high-altitude Mediterranean habitats on limestone substrate and relates to plants that grew in Sicily, Majorca, and Ibiza [13]. The EO of *T. richardii* subsp. *nitidus* studied by Bader et al. contained even 32.3% of β-bisabolene, followed by carvacrol (13.1%) and thymol methyl ether (12.4%) [14]. Thymol was present only at 1.3%. Another Sicilian EO, characterized by Castagliuolo et al. [9], contained 28.54% bisabolene in the pre-flowering phase, which was reduced to 17.91% during the flowering phase, while thymol (16.26%) and limonene (15.59%) increased significantly.

The EO of the same subspecies from the Sicilian habitat in Marettimo, studied by Llorens et al. [13], did not always have β-bisabolene as the dominant compound. It was found at the levels of 16.4–17.7%, while *p*-cymene was in the range of 15.3–20.4%, carvacrol 0–15.2%, thymol 1.5–13.9%, and γ-terpinene 2.8–7%. The same authors also described β-bisabolene-rich EOs (40.2–44.3%) from Majorca (*T. richardii* subsp. *richardii*). In that case, thymol was the second most abundant component (6.2–11.4%), and both *p*-cymene and γ-terpinene were present at low levels of 0–1% and 0–0.3%, respectively [13].

Another subspecies analyzed by Llorens and coworkers was *T. richardii* subsp. *ebusitanus* from Ibiza. It showed a transient chemotype between the sesquiterpene and phenolic types. Its EO contained a substantial amount of sesquiterpenes, such as β-bisabolene (9.6–15.9%) and β-caryophyllene (6.1–12.0%), but also phenolic compounds and related monoterpenes: *p*-cymene (7–18,6%), γ-terpinene (2.3–6.7%), carvacrol (9.8–12.8%), thymol (4.9–14.7%), and carvacrol methyl ether (13.9–18.9%) [13].

In the phenolic chemotype of *T. richardii*, we can observe EOs richer in *p*-cymene and γ-terpinene, as well as EOs dominated by thymol. The EOs of *Thymus richardii* subsp. *richardii* from two different locations in Bosnia contained predominantly *p*-cymene (35.0–67.7%), followed by thymol (2.8–21.8%) and γ-terpinene (3.5–6.6%). Similarly, EOs of *Thymus richardii* subsp. *vigoi* from Valencia contained 18.1–48.2%, 4.9–14.7%, and 2.3–6.7% of these compounds, respectively. In both cases, these EOs contained only trace or minor amounts of β-bisabolene (0.1–1.1%) [13].

In the analyzed literature, thymol has its highest concentration (34.5%) in the EO of Bosnian *T. richardii* subsp. *aureopunctatus* reported by Ćavar et al. [34], together with thymol methyl ether (23.6%), borneol (5.9%), and β-bisabolene (1.6%). Thymol was the most abundant (34.5%) in the EO of Bosnian *Thymus richardii* subsp. *aureopunctatus* in the study of Ćavar et al. [35], accompanied by other major components such as thymol methyl ether (23.6%) and borneol (5.9%). As mentioned above, the EO of the cultivar ‘Fantasia’ examined in our study contained an exceptionally high thymol level (63.69%), which has not been reported before in *Thymus richardii*. Other main constituents included *p*-cymene (5.85%), γ-terpinene (4.11%), and carvacrol (2.91%).

#### 2.2.2. *Satureja*

The following EOs of three different *Satureja montana* cultivars were studied: ‘Krymsky smaragd’, ‘Lunata’, and ‘4-18’. A total of 65 volatile components were identified and quantified using the IOFI method (Table 2, Supplementary Material S1b). The dominant compound was carvacrol, which constituted over 60% of the EO mass. All the tested cultivars can be classified as carvacrol chemotypes. The highest carvacrol content was found in the ‘Lunata’ cultivar—76.83%, while the lowest was in the ‘Krymsky smaragd’ cultivar—63.32%. These two cultivars contained very little thymol—0.46 and 0.58%, respectively. They exhibited a similar pattern of chemical composition, with the exact value of each component varying. They contained related monoterpenes: γ-terpinene and *p*-cymene, as the second and third most abundant components. In cultivar ‘4-18’, the second dominant component was thymol at a considerable level of 9.34%. Furthermore, the most abundant components were not γ-terpinene and *p*-cymene, but *cis*-thujone (2.82%) and 1-octen-3-ol (2.15%). Therefore, cultivar ‘4-18’ contains a disproportionately high percentage of thymol, camphor, and *cis*-thujone compared to the other two cultivars.

In the scientific literature, a significant chemical variability is observed when the compositions of its EOs are presented [18,36,37,38]. This variability is observed even between populations within the same subspecies [37]. The dominant compounds are usually carvacrol, thymol, *p*-cymene, γ-terpinene, and linalool. Different chemotypes are being distinguished, but in most cases, the carvacrol is the dominant one [18]. The studied cultivars present very high carvacrol contents when compared to the literature. ‘Krymsky Smaragd’ and ‘Lunata’ have already been studied by us, and very high carvacrol was detected as well, especially during the harvesting year (2019): 87% and 84%, respectively [15]. The following year, 2020, yielded less carvacrol in the EOs, namely 69% and 76%. Such consistent results suggest that high carvacrol content is a characteristic feature of those two cultivars. Yet, the actual EO composition depends on exact environmental conditions as well as the phenological phase of the harvested plant. Another consistent feature of ‘Krymsky Smaragd’ and ‘Lunata’ is very low thymol content in the EO. This study reveals only 0.46–0.58% and our last study showed 0.36–0.66% content of this monoterpenoid. Thymol is usually present in the range of 2–20%. Maccelli et al. [39] reported 7.6–16.5% in Italian and Albanian EOs, Said-Al Ahl et al. [36] found it at the levels of 2–5.9% in EOs from the Egyptian plants, and Milos et al. [38] reported values of 1.9–20.6%. However, lower thymol can also occur within the carvacrol chemotype. For example, in other work, Said-Al Ahl et al. [40] reported only 1.28% thymol in oil containing 80.39% of carvacrol, and Kustrak et al. [41] observed a thymol level of 0.58% in a sample in which carvacrol was determined at a level of 83.84%. The third studied cultivar ‘4-18’ contained a higher amount of thymol in the EO, namely 9.71%.

There is no Ph. Eur. monograph for *S. montana* EOs. There is the ISO norm 7928-1:1991, which depicts that volatile oils obtained from dried leaves of winter savory should contain γ-terpinene, *p*-cymene, linalool, 1-terpinen-4-ol, and carvacrol. The standard, however, does not provide detailed concentration ranges. It only requires their presence. Therefore, all the studied EOs from *S. montana* cultivars met the ISO standard.

#### 2.2.3. *Monarda*

A total of 62 volatile components (Table 3, Supplementary Material S1c) of EOs were identified and quantified in the EO of the studied plant from the *Monarda* genus. Both studied cultivars contained dominant thymol, which constituted almost half of the EO mass. Thus, they can be regarded as thymol-chemotypes.

The second prevalent component was varied. For *M. fistulosa* ‘Premiera’, it was *p*-cymene at the level of 16.09%. For *Monarda* × *hybrida hort*. ‘Tonya’, it was a thymol isomer—carvacrol at the level of 10.37%. The cultivar ‘Premiera’ also contained a significant amount of 1-octen-3-ol (5.73%), carvacrol methyl ether (5.41%), and γ-terpinene (5.13%), while in the cultivar ‘Tonya’, subsequent components were 1-octen-3-ol (6.33%), *p*-cymene (5.37%), and sabinene (5.08%).

*Monarda* EOs appear to be much less studied than those of the genera *Satureja* and *Thymus* [42]. Similar to those related genera, *Monarda* EOs present a variable composition of volatiles. Chemotypes rich in thymol, carvacrol, geraniol, and linalool have been described [19,20,21]. The composition of EOs of the *Monarda* L. genus is not defined in the ISO standard nor Ph. Eur. monograph. Therefore, there is no quality standard, and we can compare our results to the literature data only.

Concerning *M. fistulosa* EOs specifically, prevalent carvacrol was found by Gosh et al. at the levels of 45.7% and 71.5% in flower and leaf EOs obtained from US-grown plants, respectively [19]. Tabanca and coworkers found 39.1% of this compound in the EO from the aerial part of the plant of the same species grown in another US location [43]. Coltun & Bogdan have determined carvacrol at the level of 54.8% in the EO of this plant, but cultivated in Moldova [44]. Carvacrol also dominated at the levels of 23.7–28.2% in EOs obtained from flowers of the plant grown in Poland in two consecutive years [45]. To the thymol chemotype could be added EOs studied by Mattarelli et al. [46] (place of cultivation—Italy, thymol content—28.38–33.42%), Contaldo et al. [47] (Italy, 20.79–43.57%), Lawson et al. [21] (USA, 54.3–62.2%), Francati et al. [48] (Italy, 31.59%), and Dudchenko et al. [49] (Ukraine, 77.3–78.28%). Additionally, various other dominant compounds have been observed by different researchers for this species: *p*-cymene (32.5%), geraniol (61.8%), thymoquinone (41.3%), γ-terpinene, and α-terpineol (37.7%) [21].

The studied cultivar ‘Premiera’ represents the thymol-rich chemotype of *M. fistulosa*. Dudchenko et al. [49] provided the first description of this cultivar. They found that EO contained as much as 78.28% of thymol, followed by carvacrol methyl ether (4.8%), γ-terpinene (3.98%), carvacrol (3.58%), octenol-3 (2.11%), and *p*-cymene (2.10%). The analysis and quantification methods were not revealed. Our results confirmed that thymol is the dominant monoterpene of the ‘Premiera’ cultivar. However, its content was significantly lower—49.87%. A distinguishing feature was also the higher levels of *p*-cymene and γ-terpinene—14.08% and 5.13%, respectively. Increased levels of *p*-cymene and γ-terpinene often accompany lower thymol levels, whereas when thymol is very high, the levels of these metabolically related compounds are then lower, thus showing a negative correlation [50,51]. The outlined difference between the composition of our ‘Premiera’ EO and the composition presented by Dudchenko et al. could be explained by environmental factors. Even within the same genotype and cultivation site, EO may differ significantly depending on weather conditions or the phenological phase of the plant at harvest time. Numerous studies showed the variance of EO composition of Lamiaceae, even within the same cultivar and cultivation site, but in different years or vegetation phases [52,53,54,55,56]. In thymol-rich plants, such variation was also observed with thymol/carvacrol content peaking with different, not always consistent outcomes, but usually thymol content peaked around the flowering stages [57,58,59]. Malankina et al. [60] have studied the composition of *M. fistulosa* EO at different phenological phases across three different cultivation years and observed that its composition, including thymol content, changed significantly. At budding, thymol was at the level of 24.95%. During the mass flowering period, it dropped to 9.26% and finally reached its peak at the end of flowering (48.45%). *p*-Cymene was at quite low levels at each stage: 1.96, 4.85, and 0.9%, respectively. The latter shows how big the natural variance that occurs within the composition of EOs (phenotype) is even despite presenting the same or similar genotype.

Carvacrol methyl ether constituted 5.41% of the EO of the ‘Premiera’ variety. It was also detected at the level of 4.8% by Dudchenko et al. in the same cultivar. The second studied cultivar—‘Tonya’—contained only 0.30% of this compound. This compound seems to be characteristic of the ‘Premiera’ cultivar. On the other hand, cultivar ‘Tonya’ contained much higher thymol methyl ether than ‘Premiera’.

Cultivar ‘Tonya’ is an interspecific hybrid of two parent species: *M. fistulosa* and *M. didyma*. Similarly to *M. fistulosa*, also produces very varied EOs. Thymol-rich, carvacrol-rich, and linalool-rich chemotypes have been reported [49,61,62]. Since the EOs of the parent *Monarda* species are highly variable, we can also expect considerable variability in the EOs of interspecific hybrids. Mazza et al. studied EOs of eight samples of 5 different *M. fistulosa* and *M. didyma* hybrids and found that each of them presents a diverse composition. The first was very rich in geraniol (92.6%), the second in carvacrol (73.5%), the third in linalool (67%), the fourth in thymol (31%), and the fifth in 1,8-cineole (22%) [62]. The EO of cultivar ‘Tonya’, analyzed in this study, belongs to the thymol chemotype and contains thymol at the level of 46.70%. Compared to ‘Premiera’, it also contains significant carvacrol (10.37%), increased levels of sabinene, sabinene hydrates, and *cis*-thujone.

### 2.3. Biological Activities of EO

#### 2.3.1. Antimicrobial Activity

*Thymus*, *Satureja*, and *Monarda* EOs, which are rich in phenolic monoterpenes, present multiple biological activities, with antioxidant and antimicrobial properties being the most characteristic. An antimicrobial analysis is a key part of research on the potential use of EOs as natural preservatives or active ingredients in antibacterial and antifungal products. In our study, we have chosen three different microorganisms as the model object study: *Escherichia coli* ATCC 10536 (Gram-negative bacteria), *Staphylococcus aureus* ATCC 25923 (Gram-positive bacteria), and yeast *Candida albicans* ATCC 10231 as a representative of fungi. The solubilizer used in the study, Tween 80, did not demonstrate any inhibitory activity against bacteria and yeasts, which is a desirable result and confirms its suitability for use as an EO dispersing agent.

All the tested oils exhibited very strong antimicrobial activity in the disk diffusion method (Table 4), and the strains appeared to be extremely sensitive to the undiluted EOs. Regarding activity against *E. coli*, inhibition zones indicate very strong antibacterial activity for all the analyzed EOs compared to the standard antimicrobial drug substances—ketoconazole and gentamycin (Table 4). Among the studied EOs, the largest growth inhibition zones were obtained for the EO of *M. fistulosa*, cultivar ‘Premiera’ (ID 512), and *S. montana*, cultivar ‘Krymsky Smaragd’ (ID 516). Weak bacterial growth was observed in the presence of the cultivars ‘Tonya’ and ‘Lunata’. The *Thymus* EOs proved to be the least effective of all the tested EOs, but their growth inhibition zones were larger than the tested gentamicin doses. For gentamicin (10 µg), according to the EUCAST (European Committee on Antimicrobial Susceptibility Testing) breakpoints for a growth inhibition zone of ≥17 mm, the tested strain can be defined as susceptible [63]. Regarding the microdilution method (Table 5), also *Satureja* and *Monarda* EO were stronger and presented lower MIC and MBC than thyme oils; therefore, the observations confirmed the results from the disk diffusion method.

With respect to the antibacterial activity of the studied EOs, the results for the *S. aureus* strain were similar to those obtained for the *E. coli* strain. However, there was a difference in the MBC values, which were higher for *S. aureus*, indicating that the studied EOs were less lethal against this strain. *S. aureus* is a Gram-positive bacterium, and based on differences in cell wall structure and consensus, it would be expected to be more sensitive to the EOs [64,65]. However, our study did not show a higher susceptibility of *S. aureus* compared to *E. coli*, as observed by Borugă et al. [32]. They tested an EO from *Thymus vulgaris* cultivated in Romania (containing 47.6% thymol) against the same strains of *S. aureus* and *C. albicans*, as well as a different strain of *E. coli*, observing inhibition zones of 29, 19.4, and 19.82 mm, respectively. In our study, the inhibition zones are much larger, as follows: over 40 mm for *S. aureus* and *C. albicans*, and 33–39 mm for *E. coli*. In a similar study by Shanaida et al., *T. vulgaris* EO (47.3% thymol) caused inhibition zones of 30, 24, and 30 mm for *S. aureus*, *E. coli*, and *C. albicans*, respectively [66]. Thus, the action of the thyme EO was stronger than the effect reported by Borugă et al. [32] but weaker than that observed in our study. The EOs of thyme cultivars analyzed in this study contained a very high concentration of thymol (57.99–67.62%), which could explain the potent antimicrobial action of these oils.

Although Gram-positive (*S. aureus*) and Gram-negative (*E. coli*) bacteria typically differ in their susceptibility to antimicrobial agents; they often exhibit comparable sensitivity to thyme EO. The main reason is the high content of strong phenolic compounds. Thyme EO is rich in thymol and carvacrol, powerful phenolic molecules that disrupt cell membranes, increase membrane permeability, cause leakage of cellular contents, and inactivate enzymes. These compounds are highly lipophilic, allowing them to penetrate even the outer membrane of Gram-negative bacteria such as *E. coli*. They can integrate into the lipids of the lipopolysaccharide layer in Gram-negative bacteria, breaking down the structure of the outer membrane. Thus, *E. coli* loses much of its usual natural resistance. Thus, *S. aureus* and *E. coli* often show similar susceptibility to thyme EO because its major phenolic components are strong enough to overcome the outer membrane barrier of Gram-negative bacteria. As a result, both bacteria experience comparable membrane damage and growth inhibition [67,68,69,70].

Regarding the microdilution method, Rota et al. [71] tested the MIC and MBC values of *T. vulgaris* and *S. montana* EOs. For *E. coli*, thyme EO exhibited MIC/MBC values ranging from 0.3 to 0.8 μL/mL. For *S. aureus*, the MIC ranged from <0.1 to 0.5 μL/mL, while the MBC values were between 1 and 7 μL/mL. In the case of *E. coli*, *S. montana* EO exhibited MIC/MBC values in the range of 0.3–0.8 μL/mL, and for *S. aureus*, the MIC was lower than 0.1 μL/mL, and MBC ranged from 2 to 3 μL/mL. These results show much lower MIC/MBC values for thyme EO, indicating higher antibacterial power compared to our study (Table 5). Such a discrepancy may result from the differences in the composition of EOs and/or variations in the methodologies of microbiological analyses (different strains tested, different EO solubilizers—they used ethanol). On the other hand, for *S. montana* EOs, our MIC values were closer to those obtained by Rota et al. [71], suggesting similar antibacterial activity, although the MBC values were still higher. Nevertheless, both studies demonstrate the high antibacterial power of thyme and savory EOs.

In another study, Di Vito et al. [72] compared the MICs and MLCs (minimum lethal concentrations) of various EOs, including *S. montana*, *M. didyma*, and *M. fistulosa*, against two strains of *S. aureus* (MRSA and MSSA) and *C. albicans*. They expressed MIC values in different concentration units than those used in our study (% *v*/*v* vs. mg/mL). Therefore, we recalculated their result using an assumed density of 0.9 g/mL to make comparisons. The corresponding MIC values were estimated as follows: for *S. montana*—0.54 mg/mL (MRSA), 1.13 mg/mL (MSSA), and 2.25 mg/mL (*C. albicans*); for *M. didyma*—9.0, 4.5, and 2.25 mg/mL, respectively; and for *M. fistulosa*—0.45, 18, and 9.0 mg/mL, respectively. The MIC values obtained in our study were lower, indicating higher antimicrobial activity, especially in the case of *C. albicans*.

The tested EOs demonstrated the strongest activity against *C. albicans* fungus of all the tested strains, demonstrating their broad potential as fungicides. The measured MFC values were lower than corresponding MBC values for the studied bacteria. In the disk diffusion test, no *C. albicans* growth was observed on any plate with testes EO samples.

*Thymus*, *Satureja*, and *Monarda* EOs possess well-documented antimicrobial activity [32,65,66,73,74,75]. In fact, they, especially those from thyme and savory, are even regarded as one of the most antibacterial or antifungal EOs [64,73,76,77]. When tested in comparison with *Lavandula* EOs, they presented stronger antibacterial and antifungal activity [78]. Galgano et al. evaluated the antibacterial activity of seven different EOs (*Citrus limon*, *Pinus sylvestris*, *Foeniculum vulgare*, *Ocimum basilicum*, *Melissa officinalis*, *Thymus vulgaris*, and *Zingiber officinale*) against *E. coli* and *S. aureus*. They found that thyme EO was the strongest and, unlike other tested EOs, could totally inhibit the growth of both studied bacteria in all the tested concentrations [73].

The strong antimicrobial activity of these EOs is linked to the presence of phenolic aromatic oxygenated monoterpenes such as thymol and carvacrol, accompanied by other terpenoids. In our study, both standard substances, thymol and carvacrol, showed high efficacy in the disk diffusion method; in their presence, no growth was observed on agar plates (Table 4). However, when tested individually, thymol had higher MIC and MBC values for all the strains tested, indicating that carvacrol has stronger antibacterial activity at lower concentrations (Table 5).

The mechanism of action of thymol and carvacrol is complex and multidirectional, but it is usually mainly explained through the integration into the bacterial cell membrane, following disturbance to its normal function, leading to increased permeability and uncontrolled release of crucial cell components [79]. Regarding antifungal action, the efficacy is also explained through the destruction of the cell wall and membrane, their increased permeability, and reduced synthesis of ergosterol [64,76].

#### 2.3.2. Cellular Cytotoxic Effect of the EOs Using the Bull Semen Model

The specific physiology of spermatozoa, capable of generating motion, makes them a very useful tool or a model cell for characterizing the general toxicity of exogenous compounds in vitro [80,81,82]. Their motility is highly dependent on ATP production by mitochondria, and therefore any negative impact on these organelles (seen also as a reduction in mitochondrial membrane potential) is visible through the reduced spermatozoa motility [82,83].

**Figure 4:**
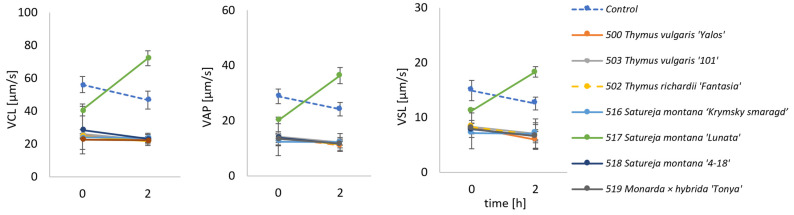
Cytotoxic effect of the EOs (at 0.4 µL/mL) during cold storage refrigeration (4 °C). Error bars represent 95% confidence intervals of the mean.

In our study, we evaluated the effect of the addition of the studied EOs on the performance and viability of bull semen. The effect of their addition (at a concentration of 0.4 µL/mL) is presented in Figure 4. The cytotoxicity of a given additive was evaluated based on the early time points only (0 and 2 h). A sharp decrease in straight-line velocity (VSL) compared to the control indicates cytotoxic effects. VSL is considered the most sensitive kinematic indicator of the loss of functional viability and the most representative criterion of sperm motility, reflecting the linear trajectory of spermatozoa [80].

The cytotoxicity of a given additive was evaluated based on the early time points only (0 and 2 h), and the evaluation of toxicity is based on time-dependent motility decline, not on the initial VSL value. At the starting point, the tested EOs showed similar or lower VSL than the control (just with the addition of Tris). Curvilinear velocity (VCL) and average path velocity (VAP) showed almost identical patterns. These results indicate cytotoxicity of the studied EO concentration at this early stage.

All the samples treated with *T. vulgaris* EO (500 and 503) and *T. richardii* (502) exhibited motility reduction, which suggests strong cytotoxicity at this concentration. This effect could be attributed to their very high thymol content (58%, 68%, and 64%, respectively) [84,85]. The *Monarda* × *hybrida* ‘Tonya’ EO (sample 519), containing 47% of thymol and 11% of carvacrol, showed a similar effect. The observed decline in the motility with the thyme EOs aligns with studies by Chikhoune et al. [86]. They studied the effect of the various doses of thymol (0.1–0.5 mg/mL) and *Thymus munbyanus* EO (0.1–1 mg/mL) on human sperm. It was observed that both studied substances reduced spermatozoa motility and their capacity to undergo hyperphosphorylation and acrosome reaction, which are important events required for eventual egg fertilization. The measured EC_50_ value of thymol was 223 μg/mL, and of EO was 781 μg/mL—higher than the value expected solely from the thymol content in the EO. The investigators suggested that the negative effects of the thymol could be partly masked or antagonized by the effects of other compounds present in the EO.

However, investigations performed by various researchers have shown that thymol can enhance sperm motility [87,88,89]. This discrepancy may be due to differences in doses, species, actual chemical composition, as well as the exact methodology used in the experiments. Vahedi et al. [87] studied the *T. vulgaris* extracts (from Soxhlet extraction) and found that the addition of extract to semen extender caused different effects depending on the doses. A concentration of 4 mL per dL of diluent (40 µL/mL) increased the total motility and percentages of viability of frozen and thawed spermatozoa compared to the control group. However, higher concentration (16 mL/dL) had a significantly negative effect on all the evaluated parameters. The researchers concluded that the addition of the extract (at a proper dose) improves the quality of the defrosted ram’s semen and suggested, based on other studies, that the protective effect can be attributed to the phenolic compounds in the extract that can protect from oxidative stress during freezing and thawing. In our study, the concentration was 0.4 µL/mL, but EO can be much more concentrated and richer in thymol compared to the studied Soxhlet extract.

*Satureja montana* cultivars ‘Krymsky Smaragd’ and ‘4-18’ (samples ID 516 and 518) showed similar toxic effects to *Thymus* and *Monarda* EOs, while cultivar ‘Lunata’ (517) presented less toxic effect at t = 0 and even some positive influence on spermatozoa motility after 2 h long storage.

#### 2.3.3. Cryopreservation Efficiency of Bull Spermatozoa in the Presence of EOs

In vitro fertilization and cryopreservation offer a chance to conceive for patients with fertility issues. Cryopreservation is also an important procedure in agriculture. It enables artificial insemination on a large scale, helps to preserve and spread favorable genetic material, and simplifies the reproductive planning of livestock. Unfortunately, storing, freezing, and thawing sperm impairs the progressive motility and viability of spermatozoa. Additionally, conventional slow cryopreservation leads to chemical and physical damage to sperm cell membranes due to the toxic effect of permeating cryoprotectants, increased lipid peroxidation, and creation of reactive oxygen species [83]. Hence, EOs, especially those exhibiting antioxidant effects, are being tested as potential semen medium additives [85,86,87,90,91].

**Figure 5:**
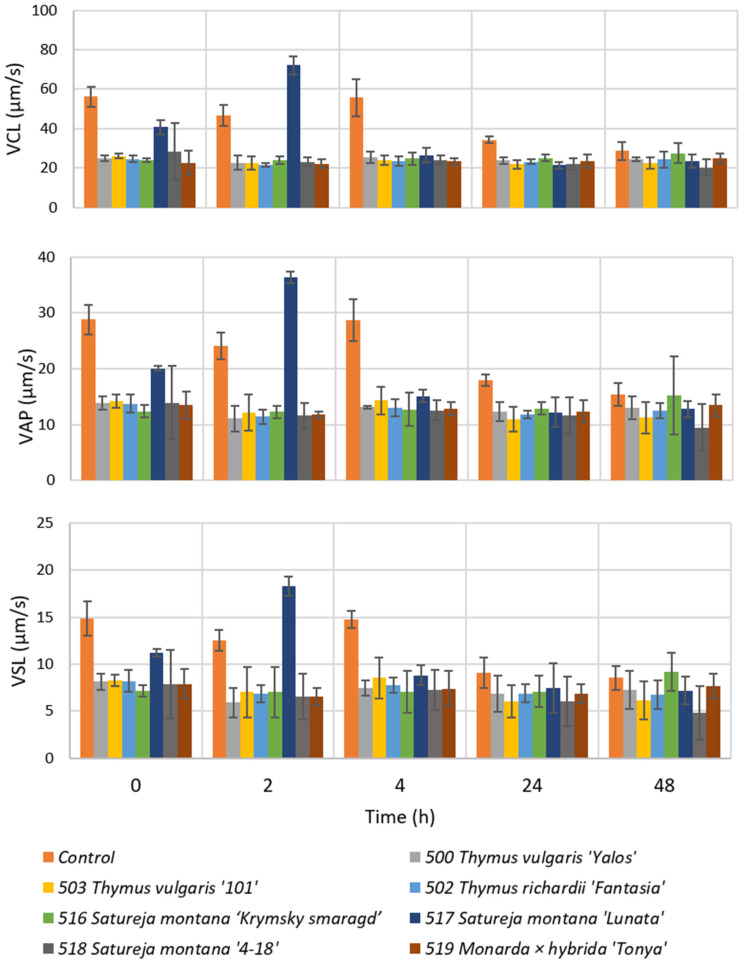
Cryopreservation (at 4 °C) of bull semen samples and temporal analysis of VCL, VAP, and VSL under exposure to different EOs at 0.4 µL/mL. Error bars represent 95% confidence intervals of the mean.

Numerous studies have identified Tris extender (spermatozoid preservation medium) as an effective cryoprotective medium for preserving the sperm viability of different animals [92,93,94]. Since *Thymus*, *Satureja*, and *Monarda* EOs are rich in phenolic compounds with proven antioxidant and antimicrobial activity, there is a potential to use them as an additive to improve cryotolerance and sperm stability and conserve the semen. Figure 5 shows the protection impact of the EOs on bull semen stored at 4 °C. At t = 0, the control group initially displayed higher sperm motility, probably due to the low exposure time, which limited the interaction and transmembrane diffusion of the EO compounds into the spermatozoa. After 48 h of cryopreservation, the highest VSL values were observed when using *Satureja montana* ‘Krymsky smaragd’ (516), indicating better motility performance after thawing compared to other treatments, and are nearly at the same level as the control, which involved just a Tris extender. This phenomenon may be due to the volatilization of volatile compounds from the EOs. Moreover, the EOs are also rich in carvacrol, a compound well-known for its protective and powerful antioxidant properties [95].

A wide range of studies have demonstrated the protective effect of EOs or their main components on various animal sperm specimens [96,97,98,99]. For example, Khnissi et al. [90] studied the addition of *T. vulgaris* EO in native and nanoemulsion form to liquid semen samples. They found that the studied EO in both forms caused a less pronounced decrease in motility than a control sample during storage. Thyme EO nanoemulsion reduced the symptoms of oxidative stress (decreased the concentration of malondialdehyde) and improved total protein and glucose levels. The researchers concluded that *T. vulgaris* in nanoencapsulation demonstrated a notable and effective role in enhancing the preservation of ovine semen [90]. On the other hand, the effect of EO is not always beneficial, depending strongly on the EO, its dose, and the animal species [80,98,99].

Our results showed that EO of *Satureja montana* ‘Krymsky smaragd’ (516) may exhibit a cryoprotective potential, likely attributable to its reactive oxygen species scavenging properties. However, the performed study was preliminary due to a limited amount of material available, and the results are non-conclusive. Further experiments are needed, especially at lower and less cytotoxic doses, to assess the potential of the studied EOs in sperm cryopreservation.

Statistical analysis revealed significant intergroup differences for all the measured CASA parameters. Two-way ANOVA showed a highly significant effect of both treatment and incubation time (from time 0 up to 48 h) on all the measured parameters (Supplementary Material S2). For example, for VSL, a strong effect of time (F_4,80_ = 36.64, *p* < 0.001) and a strong effect of group (F_7,80_ = 89.54, *p* < 0.001) were observed. Together, a highly significant group × time interaction was also detected (F_28,70_ = 18.13, *p* < 0.001), indicating that changes over time are different in different groups. Post hoc analysis showed that the control group differed significantly (*p* < 0.001) from all the treated samples. Among the tested EOs, sample 517 (*Satureja montana* cultivar ‘Lunata’) showed a significant difference (*p* < 0.001) from the other treated samples, as well as from the control. Most of the other treatments did not differ significantly from each other.

Although the statistical tests indicate significant differences in our results, a larger number of true biological replicates will be required to confirm these findings. Due to the limited availability of the studied EO material, the present experiments should therefore be regarded as a preliminary screening of biological activity.

## 3. Materials and Methods

### 3.1. Standards and Reagents

*n*-Hexane, methyl octanoate (internal standard), and an alkane standard (for LRI determination) were purchased from Merck (Darmstadt, Germany). Terpene standards were purchased at Merck, Thermo Scientific (Thermo Fisher Scientific, Waltham, MA, USA), TCI (Tokyo Chemical Industry, Tokyo, Japan), Glentham Life Sciences (Glentham, UK), and Angene International (London, UK).

Gentamycin, ketoconazole, microbiological media (MH—Muller-Hinton agar and broth), paper disks, and microbial strains were bought at BioMaxima (Lublin, Poland). Resazurin and Tween 80 from Merck were used.

Tris, fructose, and citric acid were purchased from Sigma-Aldrich (Merck), and they were of analytical grade.

### 3.2. Plant Breeding and Cultivation

The plant cultivars of three genera, *Thymus*, *Monarda*, and *Satureja*, were harvested in the Kherson region (Ukraine) in 2021. The following cultivars were bred in Kherson: ‘101’, ‘4-18’, ‘Lunata’, ‘Premiera’, and ‘Tonya’. The other cultivars were bred in the Nikitsky Botanical Garden and were introduced to the Kherson region in 2009–2010 and cultivated.

The breeding and cultivation took place in the Kherson region on introduction plots at the Research Farm “Novokakhovske” of the Rice Institute of the National Academy of Agrarian Sciences of Ukraine (NAAS), currently the Institute of Climate-Smart Agriculture of the NAAS. The research farm “Novokakhovske” is located in the first, northern agro-climatic region of the Kherson region, which is generally characterized by a temperate continental climate with a short spring, a relatively long hot and dry summer, and a mild winter with frequent thaws. The sum of active temperatures above 10 °C reaches 3200–3300 °C. Precipitation during the warm period amounts to 215–220 mm, with an annual total of 380–430 mm. The hydrothermal coefficient is approximately 0.7. The average duration of the period with positive air temperatures is 175–180 days, and the vegetation period lasts 215–225 days. Spring frosts usually cease in the third decade of April, whereas autumn frosts typically begin in the second decade of October, occasionally in late September. The Kherson region is characterized by annual droughts, about 40% of which are severe.

The plantations of aromatic plants investigated in this study were established on chernozem-like light loamy soils with a humus layer thickness of 76 cm and a humus content of 1.33% in the arable layer. Plantation management included manual weeding in the inter-rows and in the plant rows (twice a month) and drip irrigation applied as needed during the summer months (as the soil dried out).

### 3.3. Essential Oil Distillation

The EOs were obtained from the aerial parts of the plants via hydrodistillation for 45–50 min using a Clevenger-type apparatus. The EO samples were further kept in the fridge at 4 °C before analysis by GC-MS and GC-FID and then were stored in the dark and refrigerated before biological activity determinations.

### 3.4. Chromatographic Analyses

#### 3.4.1. GC-MS

The study was carried out on a Hewlett–Packard HP 6890 series gas chromatograph coupled with a Hewlett–Packard 5973 mass detector. A high-temperature-resistant ZB-5HT (Phenomenex, Torrance, CA, USA) capillary column filled with a stationary phase (5% diphenylpolysiloxane and 95% dimethylpolysiloxane) was used. The dimensions of the column were as follows: length—60 m, inner diameter—0.25 mm and film thickness—0.25 μm. The injected sample volume introduced was 1 µL. The injector temperature was at 250 °C, and the injector was set in split mode (20:1). The carrier gas was helium with a flow rate of 1 mL/min, with a flow split in the ratio of 20:1. The initial oven temperature was 50 °C, the heating rate was 3 °C/min up to 250 °C, and then it was 10 °C/min up to 280 °C. After reaching a temperature of 280 °C, the isothermal state was maintained for 5 min.

The mass detector was programmed with a 6 min analysis delay. The quadrupole temperature was set to 150 °C, the ion source temperature was set to 230 °C, and the interface temperature was maintained at 250 °C. The mass scan ranged from 30 to 550 Da.

The identification of individual components was made based on data from reference mass spectral libraries—NIST14 and Adams—additionally taking into account the linear retention indices (LRI) of individual components calculated on the basis of a mixture of alkanes. The verification of LRIs was performed using an Excel tool, Retentify [100]. For confirmation of the identifications of some peaks, reference standards were used: camphor, linalool acetate, α-terpinene, a mixture of terpineols, β-pinene, sabinene, carvacrol, and thymol.

#### 3.4.2. GC-FID Quantitation

Conditions of GC-FID analysis, along with internal standard addition and quantitation according to the IOFI-recommended practice for the use of predicted relative-response factors (RRF), were described in detail in our previous studies [101]. Tables present both chromatographic profiles according to ISO 11024, based on the peak area normalization, as well as RRF-corrected relative peak area percentages according to the IOFI-recommended method by Cachet et al. [102]. In the discussion, the latter values were referred to, as they are expected to more closely reflect the actual mass percentages.

Due to the large size of the acquired datasets, the results of three separate replicates, along with the means and standard deviation values, are presented in a separate file as Supplementary Material (Supplementary Material S1).

### 3.5. Microbiological Analyses

#### 3.5.1. General Remarks

The studies were conducted using standard strains from the American Type Culture Collection: *Escherichia coli* (ATCC 10536), *Staphylococcus aureus* (ATCC 25923), and *Candida albicans* (ATCC 10231). Microbiological analysis was performed for the EO samples, except for sample 518, due to the small amount of the research material, as well as for thymol and carvacrol standards. The positive controls (+Ctrl) were performed for gentamicin against bacteria and ketoconazole against yeast. Negative controls (−Ctrl) were performed for the Tween 80 solution and the medium.

#### 3.5.2. Disk Diffusion Method

In the disk diffusion method, thymol, carvacrol, and undiluted EOs were used for testing. Ready-to-use antibiotic disks were used as positive controls: gentamicin at a dose of 250 μg per disk and ketoconazole at a dose of 15 μg per disk. Additionally, gentamicin and ketoconazole solutions were introduced onto clean sterile paper disks at concentrations of 250 µg/mL and 15 µg/mL, respectively. Sterile paper disks were impregnated with 10 µL of the above solutions and subsequently placed onto agar media inoculated with microorganisms. A negative control analysis was performed using a 0.1% Tween 80 solution introduced into sterile paper disks.

A Mueller–Hinton medium was inoculated with 50 µL of microbial suspension (inoculum adjusted to a 0.5 McFarland standard). On a paper disk placed on the medium, 10 µL of EO/drug solution/Tween 80 was transferred. The plates were incubated for 24 h at 37 °C for bacteria and at 30 °C for yeast. The zones of growth inhibition were measured. The sensitivity of the tested strains was classified according to Ponce et al. [103] as not sensitive (diameter of inhibition zone—less than 8 mm), sensitive (9–14 mm), very sensitive (15–19 mm), and extremely sensitive (for a diameter larger than 20 mm). Analysis was performed in triplicate.

#### 3.5.3. Broth Microdilution Method

Tested concentrations of the EOs and standard compounds were as follows: 10 mg/mL, 8 mg/mL, 4 mg/mL, 2 mg/mL, 1 mg/mL, 0.5 mg/mL, and 0.25 mg/mL. The solutions were prepared from stock solutions (40 mg/mL or 4 mg/mL), and a 0.1% Tween 80 solution was used as a diluent.

A suspension of microorganisms was prepared in sterile saline with Tween 80 with an optical density of 0.5 on the McFarland scale. MH broth was used as the medium. MH broth control and negative control, which was MH broth with microbial inoculum, were performed. Additionally, gentamicin (250 µL/mL) or ketoconazole (15 µL/mL) was used as a positive control for bacteria and fungi, respectively. The plates were incubated for 24 h at 37 °C for bacteria and at 30 °C for yeast. After an incubation period, the cultures were assessed visually using an indicator—resazurin at a concentration of 0.02%. The minimum inhibitory concentration (MIC) was defined as the lowest concentration that inhibited visible growth, indicated by a color change in the initial color from purple to pink after adding the indicator.

To determine a minimum bactericidal/fungicidal concentration (MBC/MFC), 50 µL from the selected plate well was inoculated onto an MH agar plate in three replicates. The plates were incubated for 24 h at 37 °C for bacteria and at 30 °C for yeast.

After the designated incubation time, the colonies were counted, and the total number of aerobic bacteria (TBC—total bacterial count) or yeast (TYC—total yeast count) was calculated in colony-forming units per 1 mL (CFU/mL). Then, the antimicrobial effectiveness [%] of the analyzed EOs was determined using the following formula:

Antimicrobial effectiveness of a substance [%] = (C − T)/C × 100%(1)

where C—results obtained for control samples and T—results obtained for the test samples (microorganisms in the presence of EO).

The MBC/MFC value was defined as the lowest concentration at which no growth of microorganisms was observed.

### 3.6. Pre-Eliminary Cytotoxicity and Cryopreservation Studies

The cytotoxicity and cryopreservation studies were performed on bull spermatozoa following several steps. Firstly, testicles were obtained from a local slaughterhouse and transported to the laboratory under cold conditions. The epididymides were removed from the testes and thoroughly cleaned. The vas deferens and cauda epididymis were carefully separated, the blood vessels were severed, and the area was cleaned and dried.

Secondly, spermatozoa were extracted using a syringe filled with air and 1 mL of extender, which was injected into the vas deferens. The extender consisted of a buffer prepared with 30.8 g/L Tris, 17 g/L citric acid, and 12.5 g/L fructose. Each EO was prepared at a concentration of 0.4 µL/mL using Tris diluent.

Then, the collected semen was divided into portions: a control group and a treated group exposed to 0.4 µL/mL of the different EOs under study. A control sample consisted of 100 µL of sperm and 900 µL of Tris extender. Treated samples consisted of 100 µL of sperm and 900 µL of a specific EO solution in Tris extender.

All the samples were stored at 4 °C and evaluated at specified time intervals (0, 2, 4, 24, and 48 h).

At a specific time interval, the effect of each treatment on sperm motility over time was assessed using a CASA system (Sperm Class Analyzer, S.C.A. v 3.2.0, Miroptic S. L., Barcelona, Spain). For each sample, 10 μL of sperm (stored at 4 °C) was loaded into a counting chamber (10 μm depth), pre-warmed at 37 °C for 30 s, and observed under a phase-contrast microscope at 10× magnification. For each sample, three microscopic fields were analyzed to assess sperm cell parameters. Sperm motility parameters, including total curvilinear velocity (VCL, μm/s), straight linear velocity (VSL, μm/s), and average path velocity (VAP, μm/s), were measured.

### 3.7. Statistical Analysis

The statistical analysis was performed using software Statistica 13.3 (Tibco, Palo Alto, CA, USA). CASA data were analyzed using two-way analysis of variance (ANOVA) followed by Tukey’s HSD post hoc test for multiple comparisons. Statistical significance was accepted at *p* < 0.05. Graphs were prepared in Excel. Presented data points are means, and error bars represent 95% confidence intervals.

## 4. Conclusions

The GC-MS and GC-FID analysis of the studied EOs enabled their qualitative and quantitative characterization, adding to the characterization of the cultivars. The cultivars belonged to the thymol chemotype (in the case of *Thymus* and *Monarda*) and to the carvacrol chemotype (in the case of *S. montana*). *T. vulgaris* EOs did not meet the requirements of Ph. Eur. and ISO 19817 standard, mainly due to the too high levels of thymol.

All the studied EOs were rich in phenolic monoterpenes such as thymol or carvacrol. Thus, the cultivars could serve as a valuable source of these compounds, and their EOs could be used in diverse applications using the biological activities of these phenolic monoterpenoids, for instance, as natural bioactive preserving agents.

In fact, all the tested EOs showed strong antibacterial properties in both tests: disk diffusion and microdilution method for all studied microorganisms—Gram-positive bacteria (*S. aureus*), Gram-negative bacteria (*E. coli*), and the yeast (*C. albicans*). All the tested strains were extremely sensitive toward undiluted EOs. However, some differences in activity were observed, especially when diluted. Thyme EOs presented the highest MIC and MBC values, thus, the lowest antimicrobial activity.

Unfortunately, EOs also showed, at a studied concentration (0.4 µL/mL), a cytotoxic effect on bull spermatozoa, which were used as a tool for cytotoxicity assessment. However, the literature review shows that the influence of EO on sperm motility and viability is highly dose-dependent, and more detailed studies are needed to find a concentration that would be beneficial for preservation and not toxic to the cells. This aspect of the studied EOs requires more research to evaluate their potential as a natural bioactive preserving agent, whether used in the preservation of sperm or any other biological material.

## Figures and Tables

**Table 1:** Chemical composition of the studied *Thymus* EOs. The color gradient helps in a quick visual assessment of the relative content. The identity of the components in bold was confirmed additionally using reference standards. The colored background represents the relative content of the chemicals in the studied EO. * shows tentative identification.

No.	Component	LRI_exp_	LRI_ref_	Chromatographic Profile [%]				Composition Acc. to IOFI Recommended Method [%]		
				ID 500 *T. vulgaris* ‘Yalos’	ID 503 *T. vulgaris* ‘101’	ID 502 *T. richardii* subsp. *nitidus* ‘Fantasia’		ID 500 *T. vulgaris* ‘Yalos’	ID 503 *T. vulgaris* ‘101’	ID 502 *T. richardii* subsp. *nitidus* ‘Fantasia’
1	butanoic acid, 2-methyl-, methyl ester	782	777	0.15	0.19	0.18		0.22	0.28	0.27
2	*cis*-3-hexen-1-ol	854	857	0.03	0.06	0.03		0.04	0.07	0.03
3	tricyclene	927	923	0.03		0.03		0.03		0.03
4	α-thujene	930	928	0.81	0.67	0.95		0.77	0.64	0.90
5	α-pinene	938	936	0.71	0.56	0.69		0.68	0.54	0.66
6	camphene	954	950	0.81	0.53	0.67		0.77	0.50	0.63
7	1-octen-3-ol	979	980	0.93	1.01	1.06		1.04	1.13	1.18
**8**	**β-pinene**	983	978	0.25	0.23	0.27		0.24	0.21	0.26
9	3-octanone	986	985	0.06	0.07	0.07		0.06	0.08	0.08
10	myrcene	992	989	0.78	0.63	0.73		0.74	0.60	0.69
11	3-octanol	995	993	0.08	0.16	0.14		0.09	0.18	0.15
12	α-phellandrene	1009	1004	0.09	0.07	0.09		0.09	0.07	0.08
13	3-carene	1015	1011	0.07	0.06	0.07		0.07	0.06	0.07
14	**α-terpinene**	1021	1017	0.54	0.51	0.52		0.52	0.49	0.50
15	*p*-cymene	1030	1024	8.16	6.50	6.72		7.12	5.66	5.85
16	limonene	1034	1030	0.21	0.17	0.19		0.20	0.17	0.18
17	β-phellandrene	1035	1030	0.09	0.09	0.09		0.08	0.08	0.08
18	1,8-cineole	1037	1032	0.96	1.27	1.38		1.04	1.38	1.49
19	*trans*-β-ocimene	1050	1048	0.03		0.03		0.03		0.02
20	γ-terpinene	1064	1060	6.50	3.55	4.31		6.21	3.39	4.11
21	*cis*-sabinene hydrate	1073	1067	1.04	0.41	1.75		1.13	0.44	1.89
22	terpinolene	1094	1087	0.07	0.09	0.06		0.07	0.09	0.05
23	linalool	1103	1099	2.28	2.85	2.80		2.47	3.10	3.03
24	*trans*-sabinene hydrate	1105	1098	0.32	0.14	0.24		0.35	0.16	0.26
25	*cis*-thujone	1113	1105	0.11	0.09	0.11		0.12	0.10	0.12
26	*trans*-thujone	1124	1115	0.02		0.02		0.03		0.02
27	*cis*-p-menth-2-en-1-ol	1128	1123	0.05	0.06			0.06	0.06	
28	**camphor**	1155	1143	1.00	0.62	0.64		1.11	0.68	0.71
29	unidentified	1158	NA	0.47	0.49	0.64		0.59	0.61	0.79
30	lavandulol	1171	1168	0.09	0.09	0.08		0.10	0.09	0.08
31	borneol	1176	1166	2.01	1.27	1.44		2.18	1.38	1.56
										
32	terpinen-4-ol	1186	1177	1.05	0.85	0.68		1.14	0.92	0.74
33	α-terpineol	1198	1190	0.11	0.09	0.09		0.11	0.10	0.10
34	unidentified	1202	NA	0.24	0.23	0.25		0.30	0.29	0.31
35	*trans*-dihydrocarvone	1209	1201	0.06	0.06	0.07		0.07	0.06	0.07
36	carvacrol methyl ether	1249	1243	0.95	0.73	0.85		0.94	0.72	0.84
37	neral	1253	1242	0.03				0.04		
38	carvone	1261	1242	0.06	0.07			0.07	0.08	
39	geraniol	1264	1255	0.03				0.03		
40	geranial	1286	1270	0.07	0.08	0.02		0.08	0.09	0.02
41	**thymol**	1304	1290	57.39	66.99	63.25		57.99	67.62	63.69
42	bornyl acetate	1305	1284	0.53	0.43	0.33		0.63	0.51	0.39
43	**carvacrol**	1310	1300	2.74	3.38	2.89		2.77	3.41	2.91
44	thymol acetate	1360	1356	0.29		0.05		0.33		0.05
45	isobornyl propionate	1386	1383	0.04				0.05		
46	β-bourbonene	1400	1384	0.05		0.02		0.04		0.02
47	β-caryophyllene	1438	1420	3.10	1.20	1.56		2.91	1.13	1.46
48	β-copaene	1445	1433	0.03		0.01		0.03		0.01
49	α-humulene	1472	1453	0.13	0.05	0.07		0.12	0.05	0.07
50	geranyl propanoate	1476	1477	0.09		0.02		0.11		0.02
51	γ-muurolene	1490	1476	0.04		0.02		0.04		0.02
52	unidentified sesquiterpene (germacrene D *)	1498	NA	0.20		0.05		0.18		0.04
53	cubebol	1510	1515	0.03		0.02		0.03		0.02
54	α-muurolene	1513	1498	0.04		0.02		0.04		0.01
55	β-bisabolene	1518	1508	0.28		0.05		0.26		0.04
56	γ-cadinene	1530	1513	0.05	0.04	0.03		0.05	0.03	0.03
57	δ-cadinene	1537	1523	0.10	0.10	0.05		0.09	0.09	0.05
58	geranyl butanoate	1563	1563	0.02		0.02		0.02		0.02
59	germacrene d-4-ol *	1594	1574	0.03		0.02		0.04		0.02
60	spathulenol	1598	1576	0.04				0.04		
61	caryophyllene oxide	1606	1581	1.42	1.34	1.01		1.47	1.39	1.04
62	α-cadinol	1672	1652	0.09	0.00	0.00		0.09	0.06	0.05
63	amorpha-4,9-dien-2-ol	1706	1700	0.08	0.00	0.00		0.09		0.05

**Table 2:** Chemical composition of the studied *Satureja* EOs. Descriptions as in Table 1.

No.	Component	LRI_exp_	LRI_ref_	Chromatographic Profile [%]				Composition According to the IOFI Recommended Method [%]		
				ID 516 *Satureja montana* ‘Krymsky Smaragd’	ID 517 *Satureja montana* ‘Lunata’	ID 518 *Satureja montana* ‘4-18’		ID 516 *Satureja montana* ‘Krymsky Smaragd’	ID 517 *Satureja montana* ‘Lunata’	ID 518 *Satureja montana* ‘4-18’
1	2-butenoic acid, 3-methyl-, methyl ester	843	833	0.04				0.03		
2	*trans*-2-hexenal	853	853	0.05	0.02			0.05	0.02	
3	*cis*-3-hexen-1-ol	855	857	0.10	0.06			0.11	0.06	
4	1-hexanol	866	870	0.02				0.01		
5	α-thujene	931	928	1.33	0.10	0.17		1.29	0.10	0.16
6	α-pinene	939	936	0.68	0.04	0.09		0.57	0.04	0.07
7	camphene	955	950	0.16	0.02	0.04		0.13	0.01	0.03
8	1-octen-3-ol	981	980	2.86	3.66	2.70		2.37	2.99	2.20
9	**β-pinene**	984	978	0.23	0.05	0.05		0.18	0.04	0.04
10	3-octanone	988	985	0.03	0.03	0.09		0.03	0.03	0.10
11	myrcene	993	989	1.63	0.78	0.19		1.35	0.64	0.15
12	3-octanol	996	993	0.12	0.07	0.12		0.10	0.06	0.10
13	α-phellandrene	1010	1004	0.24	0.10	0.03		0.23	0.09	0.03
14	3-carene	1016	1011	0.08	0.03	0.02		0.07	0.02	0.01
15	**α-terpinene**	1022	1017	1.79	0.74	0.26		1.48	0.60	0.21
16	p-cymene	1030	1024	8.22	4.33	2.10		7.89	4.10	1.98
17	limonene	1035	1030	0.30	0.15	0.08		0.25	0.12	0.06
18	β-phellandrene	1036	1030	0.19	0.10	0.04		0.18	0.09	0.03
19	1,8-cineole	1038	1032	0.27	0.41	1.22		0.29	0.44	1.30
20	*cis*-β-ocimene	1039	1038	0.02	0.05	0.01		0.03	0.05	0.01
21	*trans*-β-ocimene	1050	1048	0.07	0.07	0.01		0.06	0.06	0.01
22	*trans*-2-caren-4-ol	1054	1154	0.03	0.10			0.03	0.10	
23	γ-terpinene	1064	1060	10.29	6.49	1.47		9.89	6.14	1.39
24	*cis*-sabinene hydrate	1074	1067	1.23	1.24	1.55		1.34	1.33	1.65
25	*cis*-linalool oxide (furanoid)	1078	1075			0.02				0.02
26	terpinolene	1094	1087	0.09	0.07	0.04		0.08	0.06	0.04
27	linalool	1104	1099	1.61	1.52	1.46		1.55	1.44	1.38
28	*trans*-sabinene hydrate	1106	1098	0.36	0.35	0.50		0.34	0.33	0.48
29	*cis*-thujone	1115	1105	0.11	0.03	2.71		0.11	0.03	2.56
30	*trans*-thujone	1125	1115	0.02		0.34		0.02		0.32
31	*cis-p*-menth-2-en-1-ol	1129	1123	0.03	0.03	0.04		0.03	0.03	0.04
32	*trans*-pinocarveol	1148	1140	0.02	0.03	0.05		0.02	0.03	0.05
33	*cis*-verbenol	1150	1144	0.02	0.02	0.05		0.03	0.02	0.05
34	**camphor**	1156	1143	0.14	0.03	2.24		0.13	0.03	2.08
35	*trans*-pinocamphone	1171	1162			0.12				0.11
36	borneol	1177	1166	0.44	0.52	0.55		0.47	0.56	0.59
37	terpinen-4-ol	1187	1177	0.84	1.01	0.99		0.91	1.08	1.06
38	*p*-cymen-8-ol	1193	1184	0.05	0.05	0.18		0.05	0.05	0.19
39	α-terpineol	1199	1190	0.08	0.11	0.15		0.09	0.12	0.16
40	*trans*-dihydrocarvone	1210	1201	0.05				0.05		
41	thymol methyl ether	1240	1234	0.03	0.02	0.38		0.03	0.02	0.41
42	carvacrol methyl ether	1250	1243	0.12	0.07	0.22		0.10	0.06	0.19
43	cumin aldehyde	1253	1238	0.09	0.23	0.24		0.10	0.24	0.26
44	linalool acetate	1259	1255	0.11	0.03	0.16		0.10	0.02	0.14
45	carvone	1261	1242	0.15	0.06	0.11		0.16	0.06	0.12
46	geranial	1284	1270			0.03				0.03
47	**thymol**	1299	1290	0.55	0.44	9.34		0.57	0.44	9.43
48	**carvacrol**	1316	1300	60.00	73.87	65.68		62.22	75.53	67.05
49	eugenol	1368	1358			0.02				0.01
50	carvacrol acetate	1380	1373	0.33	0.46	0.16		0.27	0.37	0.13
51	geranyl acetate	1386	1380	0.03	0.01	0.02		0.03	0.01	0.02
52	β-bourbonene	1401	1384	0.06	0.02	0.03		0.04	0.01	0.02
53	β-caryophyllene	1438	1420	0.56	0.22	0.33		0.60	0.24	0.35
54	β-copaene	1446	1433	0.06	0.02	0.02		0.07	0.02	0.02
55	aromadendrene	1458	1441	0.05	0.06	0.03		0.05	0.06	0.04
56	α-humulene	1472	1453	0.03	0.01	0.05		0.03	0.01	0.04
57	α-amorphene	1491	1482	0.15	0.03	0.03		0.12	0.03	0.03
58	unidentified sesquiterpene (germacrene D *)	1499	1493	0.75	0.11	0.22		0.66	0.09	0.20
59	bicyclogermacrene	1515	1494		0.04	0.10			0.05	0.10
60	β-bisabolene	1519	1508	0.54	0.25	0.13		0.58	0.27	0.14
61	γ-cadinene	1531	1513	0.13	0.03	0.03		0.14	0.03	0.03
63	δ-cadinene	1538	1523	0.19	0.05	0.04		0.21	0.05	0.05
64	hydrothymoquinone	1563	1559	0.60		0.54		0.65		0.57
65	spathulenol	1599	1576	0.11	0.00	0.00		0.12	0.44	0.44
66	caryophyllene oxide	1606	1581	0.09	0.00	0.00		0.08	0.10	0.21

**Table 3:** Chemical composition of the studied *Monarda* EOs. Descriptions as in Table 1.

No.	Component	LRI_exp_	LRI_ref_	Chromatographic Profile [%]			Composition According to the IOFI Recommended Method [%]	
				ID 512 *Monarda fistulosa* ‘Premiera’	ID 519 *Monarda* × *hybrida* ‘Tonya’		ID 512 *Monarda fistulosa* ‘Premiera’	ID 519 *Monarda* × *hybrida hort.* ‘Tonya’
1	3-heptanone	887	885	0.03			0.04	
2	α-thujene	931	928	1.10	1.00		1.05	0.95
3	α-pinene	939	936	0.36	0.34		0.35	0.32
4	thuja-2,4(10)-diene	949	956	0.05	0.04		0.05	0.04
5	camphene	955	950	0.08	0.09		0.08	0.09
6	sabinene	980	973		5.36			5.08
7	1-octen-3-ol	982	980	5.11	5.72		5.73	6.33
8	β-pinene	984	978	0.15	0.15		0.14	0.14
9	3-octanone	988	985	0.45	0.50		0.50	0.56
10	myrcene	993	989	1.12	0.89		1.08	0.84
11	3-octanol	997	993	0.37	0.42		0.40	0.45
12	α-phellandrene	1010	1004	0.21	0.14		0.20	0.13
13	3-carene	1017	1011	0.12	0.07		0.12	0.07
14	α-terpinene	1023	1017	2.02	1.21		1.94	1.14
15	*p*-cymene	1031	1024	16.09	6.22		14.08	5.37
16	limonene	1035	1030	0.52	0.33		0.50	0.31
17	β-phellandrene	1036	1030	0.17	0.12		0.16	0.12
18	1,8-cineole	1038	1032	0.11	0.66		0.12	0.71
19	*trans*-β-ocimene	1051	1048	0.02	0.02		0.02	0.02
20	γ-terpinene	1065	1060	5.34	3.79		5.13	3.59
21	*cis*-sabinene hydrate	1074	1067	1.12	1.88		1.22	2.02
22	*cis*-linalool oxide (furanoid)	1078	1075	0.08	0.06		0.09	0.06
23	terpinolene	1095	1087	0.12	0.09		0.11	0.08
24	linalool	1103	1099	0.66	0.34		0.72	0.37
25	*trans*-sabinene hydrate	1106	1098	0.24	0.71		0.26	0.76
26	*cis*-thujone	1114	1105	0.22	0.84		0.25	0.93
27	*trans*-thujone	1125	1115	0.04	0.11		0.05	0.12
28	*cis*-p-menth-2-en-1-ol	1129	1123	0.04	0.08		0.04	0.09
29	*trans*-pinocarveol	1148	1140	0.03	0.05		0.04	0.06
30	verbenol	1154	1144	0.02	0.04		0.03	0.04
31	camphor	1156	1143	0.16	0.59		0.18	0.65
32	borneol	1177	1166	0.27	0.26		0.30	0.28
33	terpinen-4-ol	1187	1177	0.94	1.10		1.03	1.19
34	*p*-cymen-8-ol	1193	1184	0.23	0.31		0.23	0.31
35	α-terpineol	1199	1190	0.30	0.26		0.32	0.28
36	*trans*-piperitol	1220	1205		0.02			0.03
37	citronellol	1235	1228	0.04	0.07		0.04	0.08
38	thymol methyl ether	1241	1234	0.12	3.19		0.12	3.15
39	carvacrol methyl ether	1250	1243	5.40	0.31		5.41	0.30
40	cumin aldehyde	1253	1238	0.39	0.24		0.41	0.24
41	thymoquinone	1263	1258	0.43	0.10		0.53	0.12
42	thymol	1303	1290	49.18	46.67		49.87	46.70
43	bornyl acetate	1305	1284	0.09	0.21		0.11	0.25
44	carvacrol	1312	1300	1.77	10.36		1.80	10.37
45	thymol acetate	1361	1356		0.07			0.07
46	eugenol	1367	1358	0.04			0.05	
47	carvacrol acetate	1379	1373		0.02			0.02
48	geranyl acetate	1386	1380	0.08			0.09	
49	α-copaene	1390	1376	0.04	0.02		0.04	0.02
50	β-bourbonene	1401	1384	0.08	0.10		0.08	0.10
51	β-elemene	1404	1390	0.03	0.09		0.03	0.09
52	β-caryophyllene	1438	1420	0.54	0.41		0.51	0.38
53	β-copaene	1446	1433	0.03	0.03		0.03	0.03
54	α-humulene	1472	1453	0.04	0.06		0.04	0.05
55	γ-muurolene	1491	1476	0.06	0.03		0.06	0.03
56	unidentified sesquiterpene (germacrene D *)	1499	NA	0.70	0.95		0.66	0.88
57	bicyclogermacrene	1515	1494	0.11	0.02		0.11	0.02
58	γ-cadinene	1531	1513	0.05	0.03		0.05	0.03
59	δ-cadinene	1538	1523	0.08	0.04		0.07	0.04
60	thymohydroquinone	1563	1559	0.84	0.79		0.98	0.91
61	spathulenol	1598	1576		0.05			0.05
62	caryophyllene oxide	1606	1581	0.10			0.11	0.11
63	amorpha-4,9-dien-2-ol	1707	1700	0.03			0.03	0.03

**Table 4:** Growth inhibition zones (in mm) in the presence of the tested substances. Legend: T—thymol, C—carvacrol, G—gentamicin, K—ketoconazole.

Sample	Essential Oils												−Ctrl		+Ctrl		
	*Thymus*				*Satureja*			*Monarda*		Standards					Solution	Drug Dose	Dose [µg]
	500	503	502		516	517		512	519	T	C						
*E. coli*	33	39	37		>40	>40		>40	>40	>40	>40		0		27	37	G250
*S. aureus*	>40	>40	>40		>40	>40		>40	>40	>40	>40		0		23	31.5	G250
*C. albicans*	>40	>40	>40		>40	>40		>40	>40	>40	>40		0		0	18	K15

**Table 5:** MIC, MBC, and MFC values (expressed as mg/mL) and antimicrobial effectiveness of a substance (in %) measured at the MIC. Legend: T—thymol, C—carvacrol, nla—lethal action was not observed within the studied concentrations.

Strain	*Thymus*			*Satureja*		*Monarda*		Standards	
	500	503	502	516	517	512	519	T	C
MIC									
*E. coli*	4	2	4	0.5	0.5	0.5	0.5	0.5	0.25
*S. aureus*	4	2	4	0.5	0.5	2	0.5	1	0.5
*C. albicans*	1	0.5	0.5	<0.25	<0.25	<0.25	<0.25	0.5	<0.25
MBC									
*E. coli*	8	8	4	4	4	4	1	4	0.5
*S. aureus*	nla	10	8	8	10	8	8	8	4
MFC									
*C. albicans*	4	4	8	0.5	4	0.5	0.5	4	4
Antimicrobial effectiveness of a substance (at MIC)									
*E. coli*	50	52	52	53	86	63	50	64	50
*S. aureus*	68	40	48	63	59	76	83	59	47
*C. albicans*	34	60	40	38	41	40	36	40	38

## Data Availability

Data will be made available on request.

## Supplementary Materials

The following supporting information can be downloaded at: https://www.mdpi.com/article/10.3390/molecules31020338/s1.

## Abbreviations

The following abbreviations are used in this manuscript:

exp	experimental
EO	essential oil
EUCAST	The European Committee on Antimicrobial Susceptibility Testing
LRI	linear retention index
ref	reference
RRF	relative response factors
MH	Muller–Hilton
MBC/MFC	minimum bactericidal/fungicidal concentration
MIC	minimum inhibitory concentration
MRSA	methicillin-resistant *Staphylococcus aureus*
MSSA	methicillin-susceptible *Staphylococcus aureus*
Ph. Eur.	European Pharmacopoeia
TBC	total bacterial count
TYC	total yeast count
VAP	average path velocity
VCL	total curvilinear velocity
VSL	straight linear velocity
